# Eye and Head Movement Recordings Using Smartphones for Telemedicine Applications: Measurements of Accuracy and Precision

**DOI:** 10.3389/fneur.2022.789581

**Published:** 2022-03-18

**Authors:** T. Maxwell Parker, Shervin Badihian, Ahmed Hassoon, Ali S. Saber Tehrani, Nathan Farrell, David E. Newman-Toker, Jorge Otero-Millan

**Affiliations:** ^1^Department of Neurology, Johns Hopkins University School of Medicine, Baltimore, MD, United States; ^2^Armstrong Institute Center for Diagnostic Excellence, Baltimore, MD, United States; ^3^Department of Epidemiology, Johns Hopkins Bloomberg School of Public Health, Baltimore, MD, United States; ^4^Herbert Wertheim School of Optometry and Vision Science, University of California, Berkeley, Berkeley, CA, United States

**Keywords:** eye tracking, smartphone, ARKit, neurologic examination, stroke, vertigo

## Abstract

**Objective:**

Smartphones have shown promise in the assessment of neuro-ophthalmologic and vestibular disorders. We have shown that the head impulse test results recorded using our application are comparable with measurements from clinical video-oculography (VOG) goggles. The smartphone uses ARKit's capability to acquire eye and head movement positions without the need of performing a calibration as in most eye-tracking devices. Here, we measure the accuracy and precision of the eye and head position recorded using our application.

**Methods:**

We enrolled healthy volunteers and asked them to direct their eyes, their heads, or both to targets on a wall at known eccentricities while recording their head and eye movements with our smartphone application. We measured the accuracy as the error between the eye or head movement measurement and the location of each target and the precision as the standard deviation of the eye or head position for each of the target positions.

**Results:**

The accuracy of head recordings (15% error) was overall better than the accuracy of eye recordings (23% error). We also found that the accuracy for horizontal eye movements (17% error) was better than for vertical (27% error). Precision was also better for head movement (0.8 degrees) recordings than eye movement recordings (1.3 degrees) and variability tended to increase with eccentricity.

**Conclusion:**

Our results provide basic metrics evaluating the utility of smartphone applications in the quantitative assessment of head and eye movements. While the new method may not replace the more accurate dedicated VOG devices, they provide a more accessible quantitative option. It may be advisable to include a calibration recording together with any planned clinical test to improve the accuracy.

## Introduction

Abnormal eye movements are observed in a variety of neurological diseases, such as stroke, ataxia, and cranial nerve damage (1). A thorough and precise analysis of eye movements can potentially provide key information regarding the affected structures (2). Examination of eye movements is quick and non-invasive and can aid the diagnosis (3). Furthermore, there are examples of eye movement examination batteries, such as the Head Impulse test, Nystagmus, Test of Skew (HINTS) exam, that have been shown to be more sensitive than MRI in diagnosing stroke in dizzy patients (4).

Despite the benefits of examining eye movements in the clinical setting, there are barriers to the widespread use of these examinations. For example, detection and interpretation of eye movements may require clinical expertise; the abnormalities may be subtle and hard to recognize with naked eyes; and sometimes quantitative measurement of eye movements is needed in order to reach a clinically meaningful conclusion (3). To overcome these barriers, video-oculography (VOG) goggles were used to objectively measure the eye movements in clinical settings (3, 5, 6). VOG has the potential to provide diagnostic clues in various clinical settings, such as emergency departments, primary care, or even patients' homes. VOG goggles are not readily available everywhere, however, due to the cost and the lack of expertise to use them and interpret their results.

Recent developments in the consumer market have introduced eye-tracking technology to common smartphones. This provides an opportunity to improve accessibility to eye movement testing technology on a broader scale. There has been more attention to the gaze tracking features of smartphones recently (7). A few studies have evaluated the accuracy of gaze tracking using smartphones and have shown acceptable findings (8–10).

In 2020, Greinacher and Voigt-Antons investigated the accuracy of eye tracking based on ARKit, Apple's eye, and face-tracking framework (11). They found that the accuracy of eye tracking based on the ARKit framework provides comparable results to methods investigated on other smartphones, tablets, and cameras (11). In a recent study, we introduced a smartphone application that quantifies one of the most common tests of vestibular function, the head impulse test also using Apple's ARKit framework (12).

In our previous study, we found that eye movement data recorded by the iPhone matched reference standard portable VOG goggles, qualitatively. However, quantitatively, the results were correlated but not exactly replicated (12). To address this observation, we need to further look into the characteristics of the recordings using the ARKit framework and the potential value of introducing a calibration produce. Calibration procedures are common in most eye-tracking devices (7) as they need to adapt to the physical characteristics of each person to produce accurate results. Thus, we need to understand whether the smartphone too could potentially benefit from a battery of tests that calibrate it prior to testing. In this study, we aimed to evaluate the precision and accuracy of eye and head position measurements using our developed application.

## Methods

### Participants

We recruited 12 healthy volunteers for this study (mean age: 41 ± 5; range: 23–69). The inclusion criteria were defined as not having known disease affecting the eye movements, being able to maintain a sitting position for the duration of the test (≈1–1.5 h), and having intact visual fields. The experiments were explained to the participants prior to testing and written informed consents were obtained. The study protocol was reviewed and approved by the local institutional review board (IRB00258938).

### Experimental Setup

Participants sat in a chair 1 m away from a central target placed at eye level on the wall. We placed targets on the wall in the horizontal plane at 5 degrees left & right from center (8.75 cm), 10 degrees (17.5 cm), 15 degrees (26.25 cm), and 25 degrees (43.75 cm). We also placed targets on the wall in the vertical plane. We placed markers on the wall in the vertical plane at 5 (8.75 cm) degrees from center, 10 (17.5 cm) degrees and 20 degrees (35.0 cm) in the upward and downward directions. The range was smaller in the vertical plane due to the inherently more restrictive nature of movements in the vertical plane vs. the horizontal plane.

We developed an application with ARKit running on an iPhone 12 pro (Apple Inc., Cupertino, CA, USA)^1^ to record both eye and head movements (12). ARKit provides continuous recordings of both eye and head positions at 60 samples *per second* using the front-facing combination of infrared and natural light cameras and sensors. We also made use of a custom timer to standardize intervals between eye and/or head movements. Lastly, we used a head-mounted laser to ensure the head was pointing at the correct target in the tests that involved head movements.

**Figure 1:**
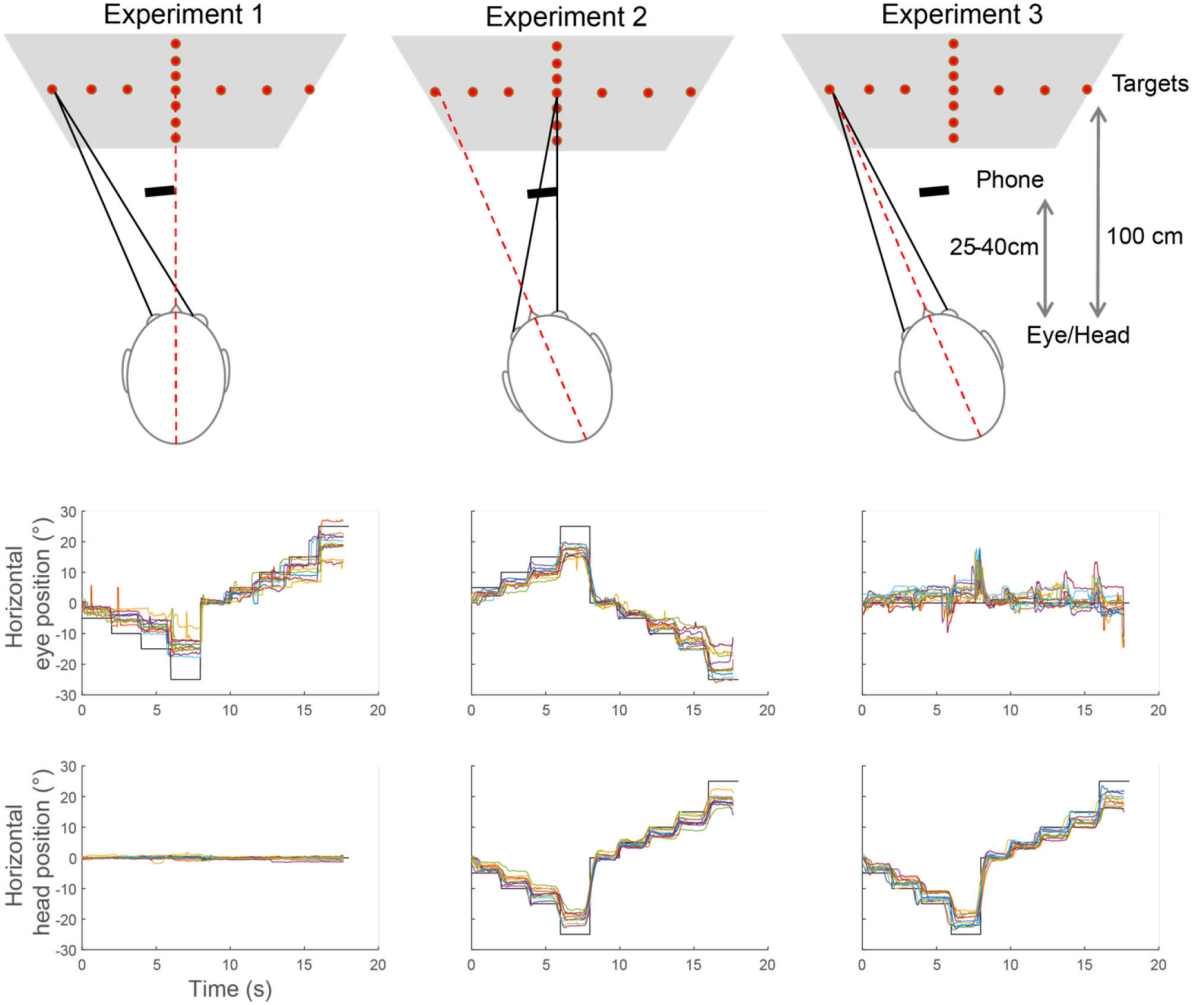
Example of eye movement recordings during Experiments 1–3. The top row shows a description of the task in each experiment. The graphs below show, for each experiment, the position of the targets (black line), the eye, and head positions measured for each of the subjects (colored lines). Each step represents the eye or head moving to the next angle.

The smartphone used to record the data was mounted on a tripod at a distance of 25–40 cm away from the patient's face—but at a slight offset so as to not obstruct the vertical or horizontal targets (Figure 1).

### Experimental Protocol

The examiner would explain the protocol to the participant and subsequently obtain consent. Then the examiner instructs the participant to do three experiments:

#### Experiment 1. Eye Only Calibration

Examiner instructs the examinee to wear head-mounted laser and ensures the laser is on and pointed on the central target. The examiner would then use the custom timer for intervals that indicate the patient should move their eyes to the next target. This timer would begin with a 3 s count down, then a chime to begin the trial with a saccade to the left (5 degrees), then another 2 s, a chime to 10 degrees, and so forth until the patient reaches the limit of the horizontal plane. Next, the patient would return to the zero-degree target before proceeding in the opposite direction, then repeat the process for the vertical plane. For our trial, we chose to begin by moving leftward in the horizontal plane, rightward in the horizontal plane then upward in the vertical plane, and downward in the vertical plane. We instructed the examinee to hold their eyes on that target until the next bell rings.

#### Experiment 2. Head Only Calibration

Repeat the process mentioned in Experiment 1, however, the head moves to the targets, while the eyes stay fixated on the central target. That is, the eyes move in the opposite direction of the head. In this experiment, the experimenter moved the head of the participant to reorient the laser toward the desired target so the participant could keep fixating on the central target.

#### Experiment 3. Head and Eye Calibration

Repeat the process mentioned in Experiment 1, however, the head and eyes move together to the targets. That is, the eyes do not move relative to the head. The experimenter moved the head of the participant to assist with simultaneous eye and head movements upon hearing the ring. Three of the twelve subjects moved the head without assistance.

### Data Analysis

The data recorded through the application was exported securely to a cloud server for post-processing and data analysis. The analysis was done in MATLAB (The MathWorks Inc., Natick, MA, USA). Blinks, squints, and other well-understood intrusive artifacts in video oculography were automatically filtered out using data streams provided by Apple, which provide information about the face. To determine a zero position, we calculated the median eye/head position of the first 2 s of the test. This was needed because of the slight misalignment between the smartphone and the central target.

Subjects were asked to look at a new target every 2 s. To measure accuracy and precision, we only used the second half of those periods to allow time for the subject to move the head and/or the eyes and reach a new static eye and head position. Figure 1 shows an example of complete recordings for horizontal eye position in Experiments 1–3.

## Results

We recorded eye movements from twelve volunteers, i.e., six women and six men, according to the methodology described previously. For each test, we calculated the accuracy and precision and plotted a chart to show the degree of error (accuracy) and the degree of variability (precision) from the true value. Across all experiments, the average percent error was 23% for eye position and 15% for head position while the precision was 1.3 degrees for eye position and 0.8 degrees for the head position. The error increased with the amplitude of the movement in all tests with an approximately linear relationship, so the percent error remained relatively constant across different positions.

**Figure 2:**
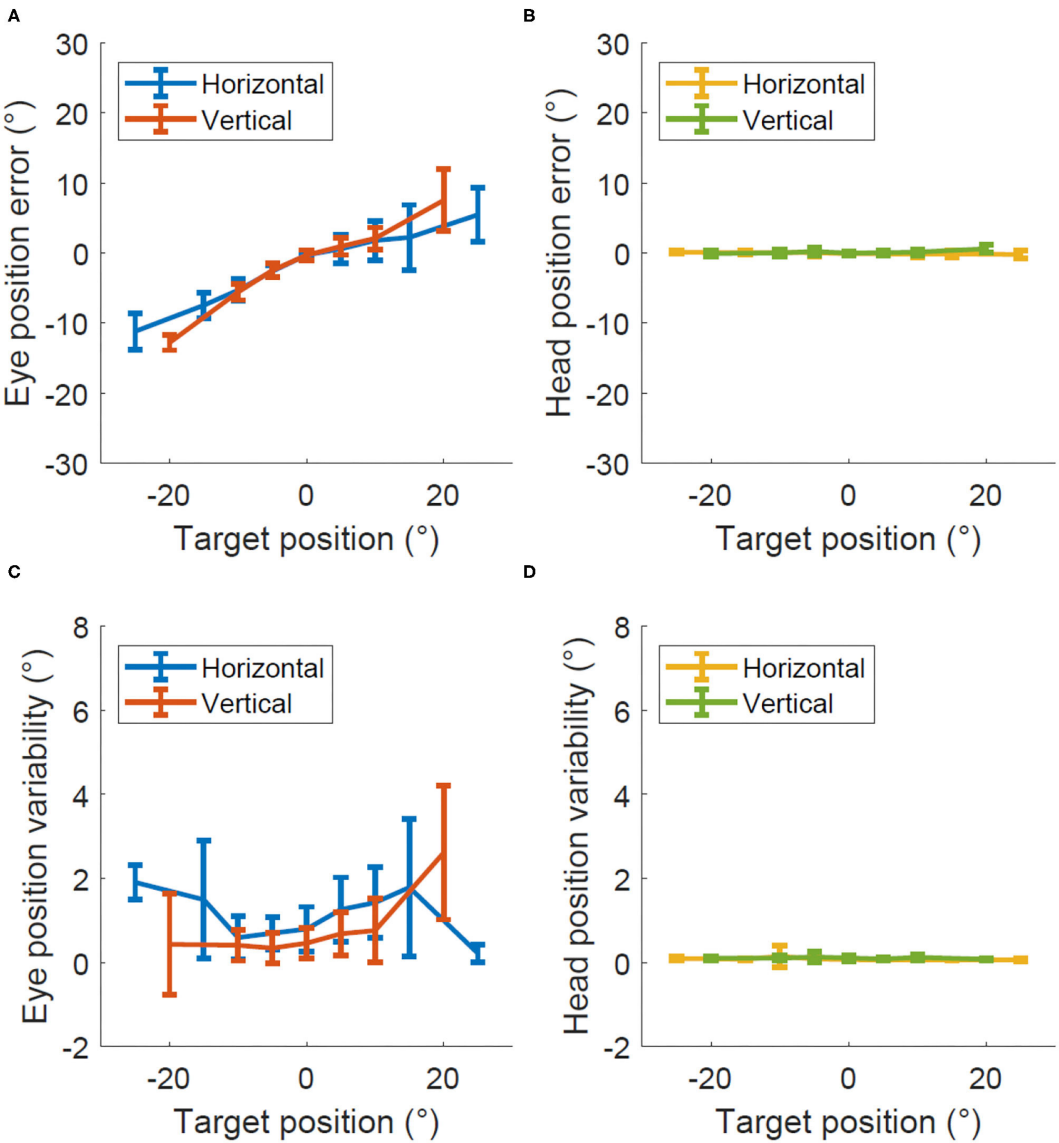
Accuracy and precision in Experiment 1, eye only calibration. Degrees of error **(A,B)** and degrees of variability **(C,D)** recorded within the eye only calibration test for both the eye **(A,C)** and head **(B,D)**.

Figure 2 shows the degree of error and variability in Experiment 1 when only the eye moved. The average percent error across all eccentricities was 33 ± 7% for horizontal eye position and 41 ± 8% for vertical eye position. The head tracking showed minimal error, accurately showing a stable head position near zero throughout the recording, 1 ± 0.2% for horizontal head position, and 0.2 ± 1% for vertical head position. The precision was 1.1 ± 0.2 degrees for eye position and 0.8 ± 0.3 degrees for head position with similar values for horizontal and vertical recordings in both cases.

**Figure 3:**
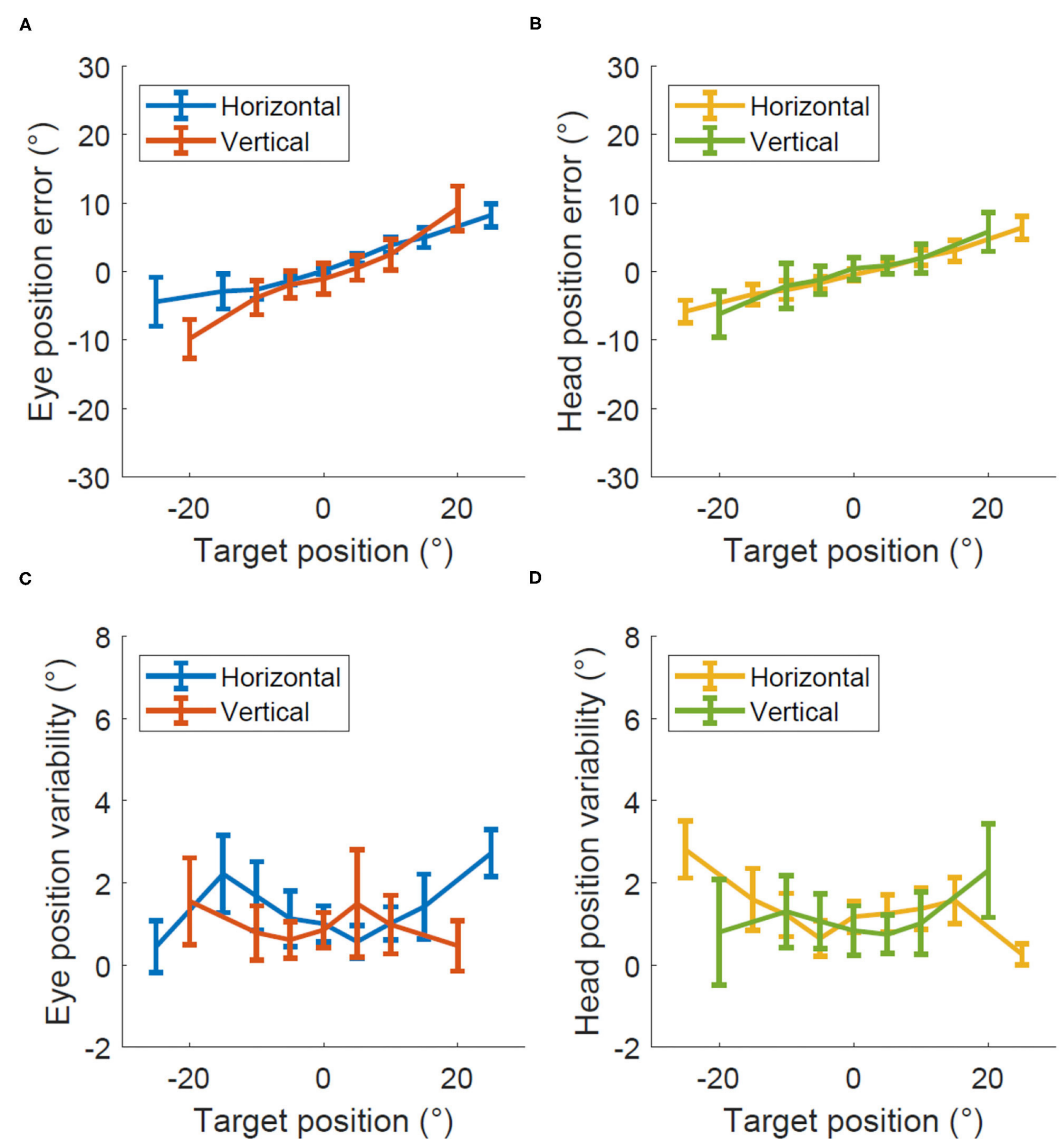
Accuracy and precision in Experiment 2, head only calibration. Degrees of error **(A,B)** and degrees of variability **(C,D)** recorded within the head only calibration test for both the eye **(A,C)** and head **(B,D)**.

Figure 3 shows the degree of error and variability in Experiment 2 when the head moved while the eyes kept fixating at the central and thus moving relative to the head. The average percent error across all eccentricities was 29 ± 3% for horizontal eye position, 34 ± 6% for vertical eye position, 23 ± 2% for horizontal head position, and 24 ± 4% for vertical head position. The precision was 1.4 ± 0.2 degrees for eye position and 1.2 ± 0.2 degrees for the head position with similar values for horizontal and vertical recordings in both cases.

**Figure 4:**
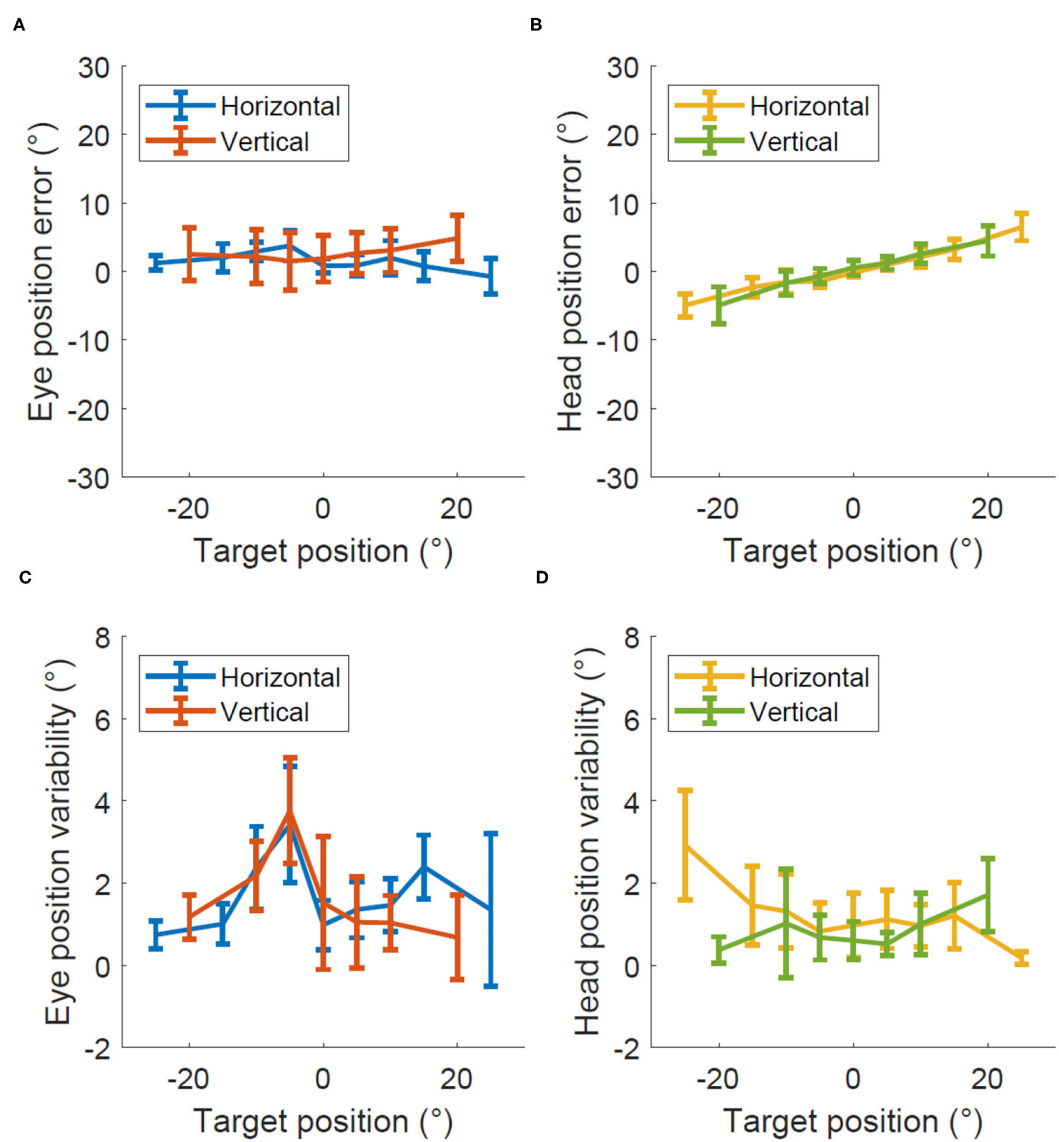
Accuracy and precision in Experiment 2, eye and head calibration. Degrees of error **(A,B)** and degrees of variability **(C,D)** recorded within the eye and head calibration test for both the eye **(A,C)** and head **(B,D)**.

Figure 4 shows the degree of error and variability in Experiment 3 when the head moved together with the eye so they both pointed toward the target and the eye did not move relative to the head. The average percent error across all eccentricities was 10 ± 10% for horizontal eye position, 7 ± 13% for vertical eye position, 21 ± 2% for horizontal head position, and 21 ± 2% for vertical head position. The precision was 1.7 ± 0.3 degrees for eye position and 1.6 ± 0.4 degrees for the head position with similar values for horizontal and vertical recordings in both cases.

## Discussion

Eye tracking enabled smartphones show great promise for the frontline assessment of eye movements in patients suffering from dizziness or other neurological disorders. In a recent study (12), we showed as proof of concept how using the application to perform the video Head-Impulse Test we could achieve a high correlation (*R* = 0.8) with measurements obtained with standard VOG devices. In this study, we focused on assessing more general metrics of data quality for eye and head position recordings. We found that the degree of error and variability increase in both eye and head movement as the eccentricity of targets increases. This is compatible with many other eye-tracking devices typically reported in the literature that have worse eye-tracking software performance as eccentricity gets larger (13). The application provided a more accurate measurement of head movements than eye movements, which we might expect due to its larger surface area and more landmarks for the smartphone to utilize when estimating where the head is facing. Also of note, the accuracy of both eye and head positions was better in the horizontal plane than in the vertical plane across tests.

Most eye trackers require a calibration before recordings. With ARKit's eye-tracking system, there is no declarative need for a calibration. Calibration is particularly useful for variations in data between individuals caused by eye shape, color, and overall compatibility with the eye-tracking software (14). Our data underscore that the smartphone shows a significant amount of error and variability between persons. We deduce then that a calibration protocol prior to testing may correct for the baseline error that each particular patient possesses. The protocol may be similar to the methodology of the experiments described here. That is, having fixed targets on a wall at known distances that subjects are asked to look at sequentially. It may also be possible that applying a general correction to all recordings produces more accurate results without the need for a calibration procedure every time. This is the aim of future studies.

Future studies must also investigate the ideal conditions for data quality obtained with the smartphone application (i.e., optimum distance from the face, optimum lighting), existing documentation alludes to certain conditions such as holding the phone anywhere from 25 to 50 cm away from the face and even though the system works in the dark, those may not be the optimal conditions.

The metrics of ARKit's ability to quantify gaze while looking at the iPhone's screen has been explored recently, with accuracy reported in the 3.18 degree range (11). This study is most closely modeled by Experiment 1, however, they differ in that the patient is looking over the screen at a target on the wall 1 m away. We found that accuracy decreased with errors of up to 10 degrees for movements of 25 degrees. This difference leads us to suggest that data may be optimal when looking at the phone's screen but deteriorates as the eye looks further away as it may have been expected since the main objective of ARKit must be tracking the eyes while looking at the device.

The next step in optimizing smartphone performance in assessing eye and head movement is to devise what such a calibration protocol may look like and measure the improvement it produces. It is of particular significance for a protocol to calibrate according to the type of examination that is planning to be measured. For assessment of the dizzy patient with HINTS battery (Head Impulse test, Nystagmus, Test of Skew), it is imperative to make use of tests that can account for nystagmus, which can be intrusive in the context of other eye movements (4, 15). It may also be possible to develop protocols that are more robust to calibration errors, such as comparing the results of the head impulse test with baseline vestibulo-ocular reflex measurements at low speed. Our previous results (12) showed a good correlation between head impulse gain measured with the smartphone and with the clinical goggles but future studies should assess in a larger population the sensitivity and specificity of the head impulse and other tests and assess if additional calibration would be beneficial. Lastly, the protocol should be streamlined for speed and practicality, as these traits are valued in the urgent assessment of the dizzy patient when ruling out stroke.

## Limitations

It remains largely unknown how ARKit quantifies eye and head movements, and thus it is difficult to interpret the variabilities in our data between persons. Rather, we focus on the utility of the results in self-calibrating the phone to obtain the most accurate assessment of eye movements going forward when compared to reference standards. Considering that ARKit is designed for the user to look at the phone, rather than a distant target, we might expect poorer performance in eye tracking. However, applying calibration protocols prior to recording may eventually overcome the poorer performance. Moreover, we should note that this technology might not ultimately provide results as accurate as standard goggles but may be of value for places without access to those goggles. Another issue common to all eye-tracking system is the potential differences in data quality when recording people from different races and ethnicities. This is something that needs to be evaluated on a larger scale with more variety of races and facial profiles.

There are a wide variety of metrics we did not test on the smartphone. Some examples include accuracy of the facial coefficients (data streams providing information on whether someone has blinked, squinted, raised their eyebrows, and so forth), accuracy of large eye movements, accuracy at varying distances, lateral displacements, different facial features, lighting arrangements, etc. There are a multitude of variables that can be explored to quantify their impact on the data and these will be a focus of future studies when determining the optimal environment for using the phone clinically.

## Conclusions

The overall accuracy of the recordings made with a smartphone was lower than other commercial eye trackers. However, all the smartphone recordings were performed without a calibration protocol. Future studies should evaluate the utility of a calibration protocol when using smartphones to assess eye movements, specially, when the movements extend well-beyond the smartphone screen. Our metrics presented in this paper justify this potential need for calibration to achieve the optimal accuracy and precision that are crucial when measuring some pathologic eye movements. However, different tests may be affected differently by different qualities of the data. For example, low accuracy may not affect detection of catchup saccades or presence of nystagmus while low temporal resolution may affect detection of catchup saccades but not measurements of VOR gain or slow-phase velocity of nystagmus.

The new method may not replace at the moment the more accurate dedicated VOG devices. However, with the potential for further improvement in both accuracy and precision, this study represents a significant step toward the smartphone's deployment in the clinic providing a new and more accessible quantitative option for eye movement recordings.

## Data Availability Statement

The raw data supporting the conclusions of this article will be made available by the authors, without undue reservation.

## Ethics Statement

The studies involving human participants were reviewed and approved by Johns Hopkins University Institutional Review Board. The patients/participants provided their written informed consent to participate in this study.

## Author Contributions

TP, AH, AS, NF, DN-T, and JO-M have contributed to designing the study. TP, SB, and NF have contributed to drafting the manuscript. TP, NF, and JO-M have conducted statistical analyses. TP, SB, AS, NF, and JO-M have contributed to interpreting the findings. AH, AS, DN-T, and JO-M have critically revised the manuscript. All authors have approved the manuscript as submitted.

## Conflict of Interest

The authors declare that the research was conducted in the absence of any commercial or financial relationships that could be construed as a potential conflict of interest.

## Publisher's Note

All claims expressed in this article are solely those of the authors and do not necessarily represent those of their affiliated organizations, or those of the publisher, the editors and the reviewers. Any product that may be evaluated in this article, or claim that may be made by its manufacturer, is not guaranteed or endorsed by the publisher.

## Footnotes

^1^About Face ID advanced Technology. *Learn How Face ID Helps Protect Your Information on Your iPhone and iPad Pro*. Apple Inc. Available: https://support.apple.com/en-us/HT208108 (accessed).

## References

[1] Leigh RJ Zee DS. The neurology of eye movements. Contempor Neurol. (2015) 5, 1–3. 10.1093/med/9780199969289.001.0001

[2] Shaikh AG Zee DS. Eye movement research in the twenty-first century—a window to the brain, mind, and more. Cerebellum. Springer (2018) 17, 252–8. 10.1007/s12311-017-0910-5 29260439

[3] Larrazabal AJ Cena CG Martínez CE. Video-oculography eye tracking towards clinical applications: a review. Comput Biol Med. (2019) 108:57–66. 10.1016/j.compbiomed.2019.03.025 31003180

[4] Kattah JC Talkad AV Wang DZ Hsieh Y-H Newman-Toker DE. HINTS to diagnose stroke in the acute vestibular syndrome: three-step bedside oculomotor examination more sensitive than early MRI diffusion-weighted imaging. Stroke. (2009) 40:3504–10. 10.1161/STROKEAHA.109.551234 19762709 PMC4593511

[5] Newman-Toker DE Saber Tehrani AS Mantokoudis G Pula JH Guede CI Kerber KA. Quantitative video-oculography to help diagnose stroke in acute vertigo and dizziness: toward an ECG for the eyes. Stroke. (2013) 44:1158–61. 10.1161/STROKEAHA.111.000033 23463752 PMC8448203

[6] Alhabib SF Saliba I. Video head impulse test: a review of the literature. Eur Arch Oto-Rhino-Laryngol. (2017) 274:1215–22. 10.1007/s00405-016-4157-4 27328962

[7] Khamis M Alt F Bulling A. The past, present, and future of gaze-enabled handheld mobile devices: survey and lessons learned. In: Proceedings of the 20th International Conference on Human-Computer Interaction with Mobile Devices and Services. (2018). p. 1–17.

[8] Kao CW Yang CW Fan KC Hwang BJ Huang CP. An adaptive eye gaze tracker system in the integrated cloud computing and mobile device. In: 2011 International Conference on Machine Learning and Cybernetics. IEEE (2011). p. 367–71. 10.1109/ICMLC.2011.6016686

[9] Huang MX Li J Ngai G Leong HV. Screenglint: Practical, *in-situ* gaze estimation on smartphones. In: Proceedings of the 2017 CHI Conference on Human Factors in Computing Systems. (2017). p. 2546–57. 10.1145/3025453.3025794

[10] Brousseau B Rose J Eizenman M. Hybrid eye-tracking on a smartphone with cnn feature extraction and an infrared 3d model. Sensors. (2020) 20:543. 10.3390/s20020543 31963823 PMC7014547

[11] Greinacher R Voigt-Antons JN. Accuracy assessment of ARKit 2 based Gaze estimation. In: International Conference on Human-Computer Interaction. Springer (2020). p. 439–49. 10.1007/978-3-030-49059-1_32

[12] Parker TM Farrell N Otero-Millan J Kheradmand A Mcclenney A Newman-Toker DE. Proof of concept for an “eyePhone” app to measure video head impulses. Digital Biomark. (2021) 5:1–8. 10.1159/000511287 33615116 PMC7879263

[13] Harezlak K Kasprowski P. Application of eye tracking in medicine: a survey, research issues and challenges. Computer Med Imaging Graph. (2018) 65:176–90. 10.1016/j.compmedimag.2017.04.006 28606763

[14] Stampe DM. Heuristic filtering and reliable calibration methods for video-based pupil-tracking systems. Behav Res Methods Instruments Comput. (1993) 25:137–42. 10.3758/BF03204486

[15] Rucker JC. Nystagmus and saccadic intrusions. Continuum. (2019) 25:1376–400. 10.1212/CON.0000000000000772 31584542

